# On Dental Students

**Published:** 1865-07

**Authors:** N. W. Williams


### Original Essays and Communications.

### ON DENTAL STUDENTS.

BY N. W. WILLIAMS, D. D. S.

Mr. President:—Having been appointed, with others, at the last meeting of this Society, perhaps it would be expected I should write upon one of the subjects named for discussion, at this meeting; but as quite a number of the members then present were appointed essayists, and doubtless will write upon these subjects, and as my mind was led in another direction and to a subject, that among the many writings of our profession is seldom if ever mentioned, much less discussed, I thought it might be profitable to consider the subject to some extent at this meeting.

My theme is—Dental Students.

The object of every true professional man should be to elevate the profession of his choice, and as the Student in every profession is the starting point of every practitioner, we should look to it that the applicant has the foundation and proper qualifications, to take rank among us, and be an honor to the profession, and not a disgrace. Every observing man among us, has long been satisfied, that had this subject received the proper attention from the masses of practitioners that it should, one of the heavy clogs upon the wheels of our young profession would long since have been removed, and our fraternity this day would have been occupying the high and exalted position all over our land, that it now occupies in some localities. Should a Literary Institution be established which admitted whoever chose to apply, without an inquiry into the moral character or preliminary education of the applicant, we can have no doubt as to the rank it would take; so with the Profession, as with an Institution of Learning, we must look well to the character and attainments of the applicant for the honors to be conferred. A writer in one of our Medical Journals, truthfully says: "The time has passed when Dentistry is to be practiced by the ignoramus. The demands of the age require education in this branch of the healing art. The progress of events, the growing importance of the art, the rapid strides of mechanical improvement, have elevated Dentistry to the position of a Profession, and imperiously demand that the recipients of its honors shall be men of science as well as art,—theoretical as well as practical."

The time has been when the Medical profession did not recognise Dentistry as a profession, for it was in its infancy, and to a great extent in the hands of the ignoramus, who followed it wholly for the money he could make out of it, and none, or very little effort was made, until such men as Prof. Harris, was raised up, to bring it from obscurity and elevate it to a profession, that would demand the respect and confidence of the age; but we are not yet clear of the evidences of the dark ages of our profession, when we have those among us that will take Students for a shorter period than any mechanic would take an apprentice to learn a trade, and after a few months in a Dental Laboratory, will thrust them out upon the public, to slay teeth on the right and left, and succeed in that locality in bringing the profession into disrepute; and should a competent dentist be so unfortunate as to locate in such a place after the "quack" has played out,

(for they will play out in every intelligent community) he will be met with amalgam fillings and poorly fitting plates, and a community decidedly opposed to filling teeth. I had a specimen apply a short time since, "to learn the dentist trade;" he said, "if I would learn him how to plug teeth, extract and such like, that he could travel through the Illinoy and make lots uv money." I informed him, that "I did not propose to be a machine to grind out quacks," and dismissed him, but he will doubtless find those who, for a good fee, will grind him out as he desires. And this is done with Dental Colleges all over our land staring them in the face.

No practitioner, who loves his profession, will receive a student unless he has the proper qualifications, and is willing to take a preliminary training, and a thorough course in some Dental College. A student taking such a course, will honor his preceptor and the profession he has adopted.

The profession in Ohio should feel proud that our noble State is honored with so fine an Institution of Learning, as the Ohio Dental College, and as a profession should sustain it with our influence and support. How many practitioners in Ohio have ever sent students to that Institution? I presume it would be safe to say not one in ten. If that Institution had relied on its native State, or the profession of the State for support, it would long since have gone under; but the profession in our sister States came to the relief by sending students,—even foreign countries have been so interested as to do their part. The sunny land of Italy had a graduate at the last commencement. If every student in Ohio were to attend our Institution, it would soon equal in importance and numbers the Ohio Medical College; and what dentist in Ohio or elsewhere who loves his profession, would not rejoice at such a state of affairs? This will be the case if we all do our duty. Let none of us receive a student unless he binds himself to take a course in some Dental College, giving our own the preference. This is our duty, we owe it to ourselves and to the world. Should this be the rule and not the exception

(as it now is), we would soon have to offer a large premium to find a Dental "quack," and be apt to fail then, for by such a course we would stop the hole where they got in.

But some may ask, are there not some "quacks" who have passed through a Dental College? We admit there is occasionally one, and will just say that a man who is a "quack" from both principle and education, is more detestable than one who has not had the advantages of learning, or the institutions of learning, and should be despised by every man who loves the profession that *he* has *disgraced* by adopting.

Let us each and all do our part to divest our profession of the least and last remains of the evidences of the dark ages of the past, and thereby elevate our profession to the high and exalted position that the age demands.

